# The Benefit of a Mechanical Needle Stimulation Pad in Patients with Chronic Neck and Lower Back Pain: Two Randomized Controlled Pilot Studies

**DOI:** 10.1155/2012/753583

**Published:** 2012-09-11

**Authors:** Claudia Hohmann, Isabella Ullrich, Romy Lauche, Kyung-Eun Choi, Rainer Lüdtke, Roman Rolke, Holger Cramer, Felix Joyonto Saha, Thomas Rampp, Andreas Michalsen, Jost Langhorst, Gustav Dobos, Frauke Musial

**Affiliations:** ^1^Chair of Complementary and Integrative Medicine, University of Duisburg-Essen, Knappschafts-Krankenhaus, Am Deimelsberg 34A, 45276 Essen, Germany; ^2^Karl und Veronica Carstens Foundation, 45276 Essen, Germany; ^3^Department of Palliative Care, University of Bonn, 53127 Bonn, Germany; ^4^Department of Integrative and Complementary Medicine, Institute for Social Medicine, Epidemiology and Health Economics, Immanuel Hospital Berlin, Charité University Medical Centre Berlin, 14109 Berlin, Germany; ^5^Department of Community Medicine, The National Research Centre in Complementary and Alternative Medicine (NAFKAM), University of Tromsø, 9037 Tromsø, Norway

## Abstract

*Objectives*. The objective was to investigate whether a treatment with a needle stimulation pad (NSP) changes perceived pain and/or sensory thresholds in patients with chronic neck (NP) and lower back pain (BP). *Methods*. 40 patients with chronic NP and 42 patients with chronic BP were equally randomized to either treatment or waiting list control group. The treatment group self-administered a NSP over a period of 14 days. Pain ratings were recorded on numerical rating scales (NRSs). Mechanical detection thresholds (MDTs) and pressure pain thresholds (PPTs) were determined at the site of maximal pain and in the adjacent region, vibration detection thresholds (VDT) were measured at close spinal processes. The Northwick Park Neck Pain Questionnaire (NPQ) and the Oswestry Disability Index (ODI) were utilized for the NP and BP study, respectively. *Results*. NRS ratings were significantly reduced for the treatment groups compared to the control groups (NP: *P* = .021 and BP: *P* < .001), accompanied by a significant increase of PPT at pain maximum (NP: *P* = .032 and BP: *P* = .013). There was no effect on VDT and MDT. The NPQ showed also a significant improvement, but not the ODI. *Conclusions*. The mechanical NSP seems to be an effective treatment method for chronic NP and BP.

## 1. Introduction

Chronic pain syndromes affecting the neck and lower back are very common and thus clinically relevant [1–7]: the lifetime prevalence for neck pain (NP) is approximately 48–66% and for lower back pain (BP) 51–84%. Of these patients, 9–18% suffer from severe chronic NP and 15–37% from severe chronic BP [1–4, 6–9].

Both syndromes therefore have already a high socioeconomic relevance and the incidence is still increasing. It is estimated that the total costs of low-back pain in the USA exceed 100 billion US $ per year, including the direct costs for treatment and the indirect costs for work absenteeism [10, 11]. The total costs of neck pain in The Netherlands in 1996 were estimated to be 686 million US $ [12]. 

Chronic pain syndromes of the back such as lumbago and/or neck pain are essentially characterised by muscle pain and thus relate to deep somatic pain. Like visceral pain, deep somatic pain is dull, difficult to localize (for an overview see [13]), and difficult to treat. 

Furthermore, the complaints can be very obstinate. Therefore, treatment remains challenging. The underlying cause is often not easily detected, as there is a mismatch between the patient′s complaints and suffering and the “objective” diagnostic results (e.g., MPI, CT, clinical examination) [14–16]. This particular clinical situation is commonly referred to as “chronic pain disorder” and requires a special approach to treatment. 

Since BP and NP are usually of deep somatic origin, it seems reasonable that, in order to treat these pain syndromes, naturopathic therapists often apply methods that specifically deform and manipulate somatic body structures, such as subcutaneous tissue, skeletal musculature, and fascias. Consequently, the American National Centre for Complementary and Alternative Medicine subsumes these therapies under the superordinate concept of “Manipulative and Body-Based Practices” (http://nccam.nih.gov/health/whatiscam).

Well-known examples of such treatments are, for example, massages, cupping, and the rhythmic massage of anthroposophic medicine [17]. Until now there is limited evidence that manual therapies can be effective treatment for chronic BP and NP [18, 19], nonetheless CAM receives increasing attention in the treatment of both chronic pain conditions [20–22]. 

One self-administered device used for the treatment of NP and BP is the needle stimulation pad (NSP). The NSP—with more than 1000 sharp, but nonpenetrating plastic needles, is usually placed on soft ground and the patient lies for a limited time period on top of the mat with the uncovered, painful part of the body. It is very popular in some countries (Scandinavia) and is distributed under numerous names [23–25]. Those devices all have in common that they contain a flexible material, mostly cloth or soft plastic. On this pad sharp plastic spikes are affixed. There is a resemblance to the well-known bed of nails of indian sadhus or wise men. In the 1980s, a modern version was reinvented in Russia to serve as a kind of self-acupuncture device [23, 25]. Since then, multiple pads are widely used either as a tool for well-being or as a medical device. Indications include pain disorders, especially pain of the lower back, neck and headache, but also sleeping disorders, diseases of the gastrointestinal tract or just for relaxation [23, 25]. Mostly the pad is applied directly at the painful area, but some pads are bigger in size and are used underneath the lying whole body. These mats are often used for relaxation. The NSP is mostly self-administered without the survey of a therapist, therefore little is known about the different practices of application. Furthermore, the treatment outcome of the NSP and the mechanisms of action remain unclear. However, due to the intense mechanical stimulation of the skin, the subcutaneous tissue, and muscles induced by the needle stimulation, it can be speculated that its mode of action is similar to other complementary and alternative medicine (CAM) manual and physical therapies, namely, via direct mechanical stimulation of the skin and the subcutaneous tissue [26, 27]. The NSP likely activates skin mechanoreceptors and even nociceptors, a process that might affect the transmission and processing of sensory information to the spinal and supraspinal level [27]. 

A recent study investigating the effect of a NSP in healthy young volunteers showed substantial effects on cardiac autonomic responses and reactions of the sympathetic and parasympathetic nervous system, such as self-rated relaxation, blood pressure, heart rate, heart rate variability, and back temperature [25]. In contrast to the data presented here, the pad was bigger in size and covered a larger area of the back.

Quantitative sensory testing (QST) is a well-established procedure and is predominantly used for the analysis of the somatosensory phenotype of patients suffering from neuropathic pain [28, 29]. However, subtests of the test battery, especially the pressure pain threshold (PPT), have been utilized as outcome measures in treatment studies on chronic pain [30–41]. 

The aim of the two present pilot studies was to investigate whether a two-week treatment period using the NSP changes the pain levels of participants with chronic NP and lower BP. Furthermore, possible treatment effects on mechanical sensory thresholds, in particular mechanical detection (MDT), pressure pain (PPT), and vibration detection (VDT) thresholds were investigated. The whole QST-protocol would have consumed up to an hour per location, so that the entire protocol was considered to be too time consuming for a pilot study. Therefore, these particular QST subtests were chosen for pragmatic reasons, because they are indicative for the two different pathways, which can be considered to play a role. VDT and MDT are indicative for the lemniscal and PPT for the spinothalamic pathway (for review see [42–44]).

## 2. Methods

### 2.1. Participants

Inclusion criteria were age between 18 and 75 and nonspecific NP or lower BP for at least three consecutive months. The patients were excluded if they reported radicular pain, showed neurological symptoms suggesting a disc prolapse, had a vertebral column surgery less than 12 months prior to the study, a chiropractic manoeuvre or infiltration at the area treated 4 weeks prior to the inclusion in the study, or a congenital deformation of the spine. Further exclusion criteria were an insulin-dependent diabetes mellitus, dermatological diseases or skin changes at the treated area, severe mental illness that required medication, a known tendency for haemorrhages, current anticoagulation, or corticosteroid medication. Pain medication with the exception of corticosteroids as well as physiotherapy were allowed, if the treatment regimen was not altered for four weeks prior to the study and continued throughout the study. Other related physical or manual therapies like acupuncture, cupping, TENS were not allowed. Participants were recruited using flyers, by announcement through the homepage of the Chair of Complementary and Integrative Medicine, Kliniken Essen-Mitte, Germany, and through a local newspaper article. Study applicants were screened twice, first by a standardized telephone interview and second by the study physician, who examined the patient at their first appointment. This “on site” assessment during the first visit was performed to ensure patient safety. All inclusion and exclusion criteria were checked again, a careful patient history was taken, and a thorough physical examination, including neurological examination, was performed to rule out a clinical suspected spinal alteration, for example, discus prolapse. All participants provided written informed consent before participating in the study.

### 2.2. Study Design

The study was planned and conducted in compliance with the Declaration of Helsinki (World Medical Association) and the Guidelines for good clinical practice. The protocols were approved by the institutional review board of the University of Duisburg-Essen Medical Institutions, Germany (no. 08-3767 for BP and 08-3768 for NP). Both studies were carried out independently but according to a similar protocol. They were conducted as two-week open randomized controlled pilot trials at the Department of Complementary and Integrative Medicine Kliniken Essen-Mitte, Essen, Germany from October 2008 to May 2009. 

At the first examination, a medical history was collected and each participant was thoroughly examined. Subjective assessments on pain and function were taken, and sensory thresholds were determined. Subsequently the participants were randomly assigned to a home-based treatment either (TG) or waiting list control group (WL). The participants in the TG were thoroughly instructed how to use the NSP. All patients were asked to document medication and physiotherapy treatments in a treatment diary. Fourteen days later the evaluation of NRS ratings, the questionnaire and the sensory examination were repeated for both groups. After the second examination day, patients of the WL received the NSP.

### 2.3. Treatment Allocation

In order to rigorously ensure that at the time of enrolment, medical examination, and baseline measurement the group allocation was unknown to everybody involved in the study, randomization was performed after completion of the first (baseline) measurement. The randomization procedure itself was performed as a lottery, without stratification or sequencing. The “lottery tickets” consisted of the exact number of “treatment” or “control” tickets needed, hidden in sealed, nonmarked opaque envelopes. These envelopes were prepared before the enrolment started and kept in a “lottery box”. The envelopes were manually shuffled before each draw and the patients picked an envelope after the completion of the first measurement.

### 2.4. Intervention

**Figure 1:**
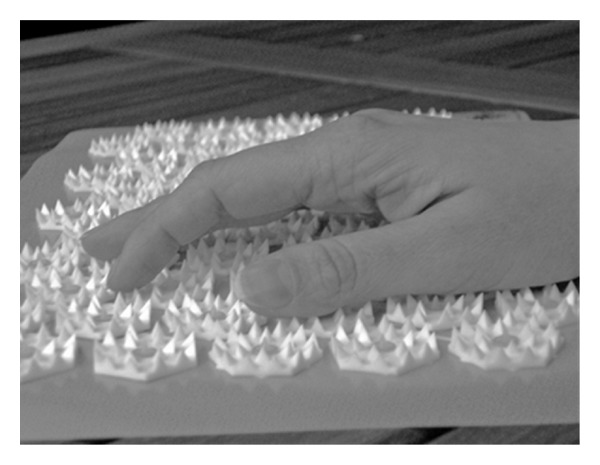
Needle stimulation pad.

The treatment consisted of a home-based, self-administered intervention with the mechanical NSP (see Figure 1). 

It is a medical device, which has been applied for years by patients with diverse pain syndromes. Different versions of this pad are available in various sizes. The same NSP was used in both studies: a 22 × 33 cm plastic mat featuring 60 hexagonic plastic discs. On each of these discs, 19 spikes are affixed with the distance between the 1140 spikes being 5 mm (Zhencidian pad, CMP Chinese Medical Products Trading GmbH, Austria). 

Participants were instructed to apply the NSP over a period of 14 days once daily according to the following procedure: in a first step, the patients were instructed to press both hands (only NP study) or both feet (only BP study) for 10 minutes on the mat while sitting on a chair. The protocol is to treat the hands of patients with neck pain and feet of patients with lower back pain, respectively, prior to the actual painful area derived from clinical experience. The protocol represents the way the NSP is applied in our clinic. It was recommended to place the NSP afterwards on a soft base, for example, the bed and to lie on top of the mat with the uncovered painful part of the body (neck or lower back, resp.). The patients were also instructed to place a towel or a bedroll underneath the neck, if the neck was to be treated. The participants were informed that the first 2 to 5 minutes could be painful. A daily treatment time of about 30 minutes was recommended for the painful part of the body. 

### 2.5. Subjective Measures

A numerical rating scale (NRS) ranging from 0 = no pain to 10 = worst pain imaginable was used [45, 46]. Furthermore, the Oswestry Disability Index (ODI), which measures the disability of patients with lower BP, was utilized [47–49]. The ODI score ranges from 0 to 100. A modified Northwick Park Neck Pain Questionnaire (NPQ) (which is scored out of 32 or 28 for noncar drivers and is presented as a percentage) was used to assess the severity of chronic NP. The NPQ score ranges from 0 to 100, with higher scores indicating higher pain and lower function [50].

#### 2.5.1. Mechanical Sensitivity to Superficial versus Deep Stimuli

Sensory testing was performed in a quiet room with constant room temperature (approx. 82–84°F) and air humidity (approx. 75%) and included the MDT, PPT, and VDT according to the quantitative sensory testing protocol [28, 29]. In order to eliminate test-retest bias due to different assessors, only one assessor was assigned to each of the studies. Both assessors were thoroughly trained to perform the procedure.

All sensory tests were conducted at the point of maximum pain and 10 cm caudal paraspinal (for NP patients) or 10 cm cranial paraspinal (for lower BP patients), respectively. Furthermore, sensory tests were conducted at two control sites apart from the painful area, the right hand, and foot, serving as measures of intraobserver reliability.

#### 2.5.2. Mechanical Detection Threshold (MDT)

MDT was tested with a standardized set of von-Frey-filaments (The SenseLab Aesthesiometer, Somedic, Sweden). Von-Frey-filaments are nylon filaments with a rounded tip; affixed in a rectangular manner at a handlebar. They are pressed perpendicularly with constant force onto the skin surface (paravertebral, hand dorsum, and foot dorsum) until the filament bends. Using the method of limits, a psychophysical method to approximate thresholds, five threshold determinations were made, each with a series of ascending and descending stimulus intensities. The test always started with a von Frey filament that was clearly sensed by the test person. In the next step, the examiner took the next smaller filament. This procedure was repeated until the test person was unable to sense a filament. Then this particular filament was annotated as the first subliminal stimulus. Now the test was continued in ascending order, and the next bigger filament was taken again until the test person could feel a filament. This is the next value above the threshold. The final threshold is represented by the geometric mean of these 10 values.

### 2.6. The Final Threshold Was the Geometric Mean of These Five Series

#### 2.6.1. Pressure Pain Threshold (PPT)

PPT was determined by means of an algometer (Somedic, Sweden) with a probe area of 1 cm^2^ using three series of ascending stimulus intensities (slowly increasing ramp of ~20 kPa/s) at a muscular area (paravertebral muscles, thenar eminence and foot dorsum over the musculus extensor hallucis brevis). The patient was instructed to press a button, as soon as this pressure was perceived as being painful. The final threshold was the arithmetic mean out of all 3 measurements.

#### 2.6.2. Vibration Detection Threshold (VDT)

VDT was measured with a Rydel Seiffer tuning fork (64 Hz, 8/8 scale) which was placed over a bony prominence (processus spinosus, processus styloideus ulnae, and malleolus internus). After the tuning fork has been induced into vibrations it was placed over the tested area. The tested person was asked, if he sensed the vibration and should indicate, if the vibration had stopped. The particular value was read out of the 8/8 scale and the final threshold was the arithmetic mean out of 3 subsequent descending measurements.

The control group was offered the same NSP postintervention.

## 3. Data Analysis

These trials were pilot trials not only designed to explore the effectiveness of the NSP but moreover to generate hypotheses about the potential mechanisms of action. Thus, only patients giving a complete set of data were analysed per protocol, and missing values were not imputed. 

According to the Quantitative Sensory Testing (QST)-protocol [28, 29] PPT and MDT data were transformed logarithmically before statistical analysis, VDT data were analysed as raw data. NPQ and ODI scores were calculated and expressed as percentages (ranging from 0 to 100). 

Each outcome parameter was analysed by a univariate analysis of covariance, modelling treatment as a fixed factor, and the respective baseline value as a covariate. Differences between treatment groups were tested by two-sided *t*-tests within these models. All analyses were conducted with the SAS statistical software (release 8.2, SAS Institute, Cary NC, USA). *P* values < .05 were regarded as statistically significant.

## 4. Results

**Figure 2:**
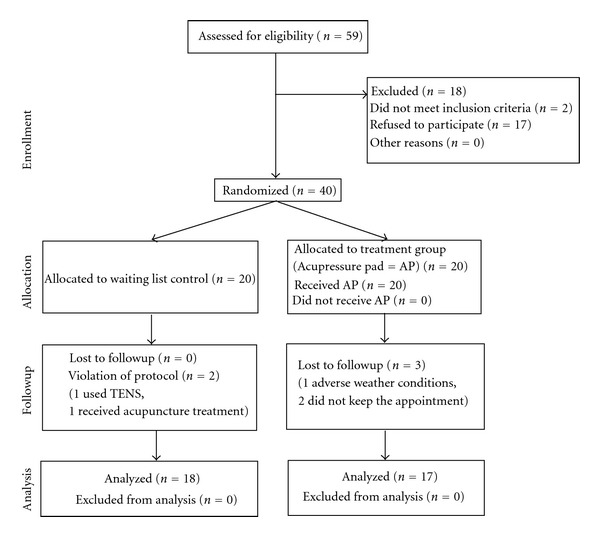
Consort E-flowchart: neck pain study.

**Figure 3:**
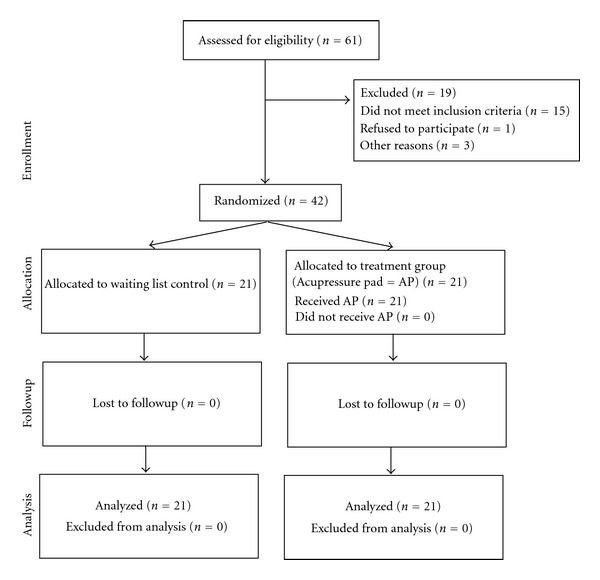
Consort E-flowchart: back pain study.

Forty patients were included in the NP and 42 patients were included in the BP study (see flow charts Figures 2 and 3). 

Of the participants recruited for the NP-study 3 participants were lost to followup due to reasons not related to the study. Moreover, 2 participants, both from the control group, violated the treatment protocol. One began a TENS treatment during the study and the other was hospitalized with severe NP and shoulder pain and received repeated acupuncture treatments. Of the 42 patients recruited for the BP-study, nobody was lost to followup or violated the protocol. Most patients in both studies were females, the average age was 46.1 (±11.3) years in the in the NP study and 63.9 (±11.1) years in the BP study (Table 1).

While there were no baseline differences in the BP study, baseline difference for NRS ratings and NPQ scores occurred in the NP study (Table 2).

Moreover, gender was not equally distributed across groups: 85% of the patients in the treatment group were females compared to 100% in the control group (Table 1). 

### 4.1. Pain and Disability

**Figure 4:**
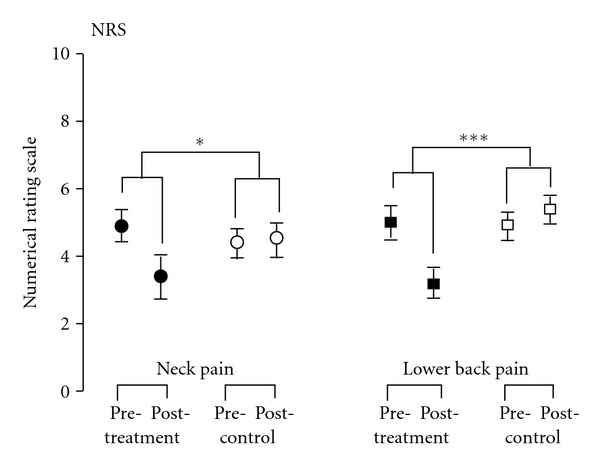
Numerical rating scale (mean ± SEM) of patients with chronic neck pain and chronic lower back pain at baseline (pretreatment) and after 2 weeks (posttreatment). Patients in the neck pain treatment group (filled circle) as well as in the lower back pain treatment group (filled square) showed a significant treatment effect compared to the controls (open symbols). *P* values are indicated as *P < .05, ***P < .001.

In both studies, treatment reduced the NRS pain ratings significantly compared to the control. In the NP study, the estimated group difference was *d* = −1.6 pts; with 95%-CI: −2.8 to −.3 (*P *= .021) and in the BP study it was *d* = −2.3 pts with 95%-CI: −3.2 to −1.3 (*P* < .001) (Figure 4, Table 2).

**Figure 5:**
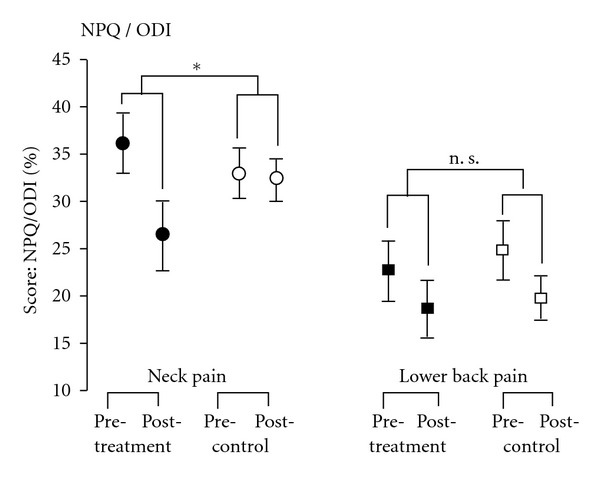
Scores of the Northwick Park Neck Pain Questionnaire (NPQ) and Oswestry Disability Index (ODI) (mean ± SEM) of patients with chronic neck pain and chronic lower back pain at baseline (pretreatment) and after 2 weeks (posttreatment). Patients of the neck pain treatment group (filled circle) had significantly lower neck-pain-related disabilities after 2 week treatment compared to patients in the waiting list control (open circle). No such effect could be detected in the ODI in lower back pain patients (filled square = treatment group, open square = control group). *P* values are indicated as *P < .05, n. s. (no significance).

NP-induced disability was significantly improved after treatment compared to the control group by *d* = −7.4 NPQ score points (95%-CI: −13.7 to −1.1, *P* = .028) (Figure 5, Table 2), while there was no group effect in the BP study (difference in the ODI score: *d* = .4; 95%-CI: −4.8 to 5.6, *P* = .878) (Figure 5, Table 2). 

### 4.2. Mechanical Pain and Detection Thresholds

Test-retest correlations in the control areas (hands and feet) were high and statistically significant, indicating good reliability and accuracy (see Table 3).

**Figure 6:**
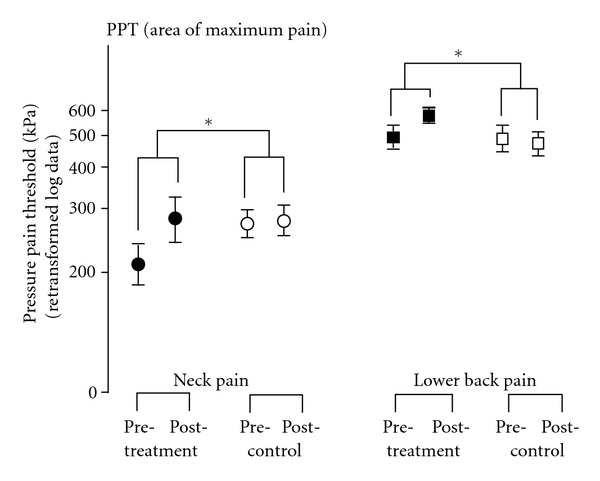
Pressure pain thresholds (mean ± SEM) at the area of maximum pain of patients with 413 chronic neck pain and chronic lower back pain at baseline (pretreatment) and after 2 weeks 414 (posttreatment). Patients in the neck pain treatment group (filled circle) as well as in the 415 lower back pain treatment group (filled square) showed a significant treatment effect 416 compared to controls (open symbols). *P* values are indicated as *P < .05.

A significant group difference was seen for NP (*d* = .106; CI: .013 to .198, *P* = .032) and BP (*d* = .082; CI: .021 to .144, *P* = .013) for log PPT at the point of maximum pain (Figure 6, Table 2), while at 10 cm distance from pain maximum this was the case for BP only (*d* = .076; CI: .006 to .139, *P* = .038). 

**Figure 7:**
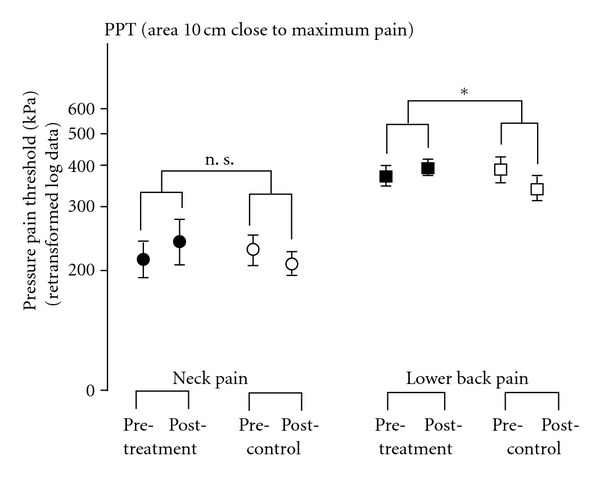
Pressure pain thresholds (mean ± SEM) at the area 10 cm close to maximum pain of patients with chronic neck pain and chronic lower back pain at baseline (pretreatment) and after 2 weeks (posttreatment). Only patients in the lower back pain treatment group (filled square) had a significant treatment effect compared to patients in the control group (open square). No significant effect could be found in the neck pain group (filled circle = treatment group, open circle = control group). *P* values are indicated as *P < .05, n. s. (no significance).

There was, however, a tendency for greater pain threshold to pressure-induced pain at 10 cm distance from maximum pain in the NP study (*d* = 0.85; CI: −.002 to .171, *P* = .064) (Figure 7, Table 2).

No statistically significant effects between patients and controls were seen for PPT at the control areas, as well as for MDT and VDT in general (Table 2). 

### 4.3. Adverse Effects

Besides the discomfort or pain when lying on the pad, especially at the first minutes of the first sessions, no adverse effects of the mechanical NSP were reported.

## 5. Discussion

The outcome of the two studies presented here further supports the usefulness of the NSP as a representative of naturopathic therapies in the treatment of chronic pain syndromes. 

To our knowledge, the two studies are the first to systematically investigate the outcome of the NSP as a therapeutic agent in two highly prevalent chronic pain syndromes. The results show that the NSP significantly reduced pain ratings in patients suffering from chronic pain of the neck or the lower back. Pre- to posttreatment decreases in the NRS of 30% in the NP study and 36% in the BP study are in the range of a moderately important clinical difference [51, 52]. The effect was robust and stable even after a comparatively short treatment period (as daily treatments for 2 weeks). At least in NP, as shown in the NPQ, the treatment improved physical functioning and thus reduced the NP-related disability. Why the ODI was slightly worse than at baseline remains unclear. 

Chronification of pain may originate at the level of the nociceptor, the spinal cord, or the brain (e.g., [17, 26]). Although these mechanisms cannot be separated in a chronic pain condition, different treatment strategies may have their focus on different levels of the pain process. 

It is assumed that hyperalgesia is associated with the hyperexcitability of central neurons in the spinal cord [53, 54]. As hypersensitivity is caused by peripheral and central sensitization [55, 56] these changes are by far not static but build the matrix of the dynamic receptive field plasticity [57]. The peripheral sensitization is thought to be mediated by an axon reflex, which sensitizes the receptors. Central sensitization occurs with a long lasting enlargement of receptive fields and recruitment of silent nociceptors. The threshold for afferent stimuli is decreased, caused by permanent neuroplastic changes in the spinal cord [57]. These mechanisms are hypothesized to be involved in the development of a chronic pain disorder even when the initial injury or inflammation is no longer present [58]. The presence of widespread sensory hypersensitivity is linked to central hypersensitivity, augmented central pain processing [59], or decreased descending control of pain [60].

 Naturopathic treatments that induce controlled but clearly visible injuries of the skin, such as, for example, gua sha massage or wet cupping, markedly alter the nociceptor environment. It can be assumed that these injuries, which are often clearly visible for several days, lead to increase firing of the nociceptive fibres in the affected regions [61].

Such an increased firing for several hours up to days may directly influence the receptive fields of spinal neurons in the affected region. If this is the case, these treatments are likely to induce specific, demonstrable effects on the somatocutaneous or viscerosomatic projection areas. Such effects can be demonstrated by measuring the patients' pain thresholds. (For a comprehensive test battery on sensory testing, see the QST-protocol [28, 29]). Interestingly enough, pressure pain thresholds were increased at the point of maximum pain in both studies, reflecting that treatment has at least locally (peripherally) reduced hyperalgesia to blunt pressure in both syndromes. In the BP study, reduction of deep pain sensitivity was also present remote from the most painful area. These findings suggest that part of the treatment effect seems to be due to changes of nociceptive processing in the spinothalamic tract as well as at the level of the central nervous system. 

Because there were no relevant differences in VDT and MDT, a possible involvement of the lemniscal tract is not likely. Larger studies including a nontreatment control group, a healthy control group, and including further QST-tests as well as the assessment of both body sides will reveal more conclusive evidence for the pathway in question.

Previous studies in which various forms of cupping therapy, for example, dry cupping [40], wet cupping [38], and pulsating cupping [39] have been examined with QST in patients with neck pain showed similar results: improvement in pain ratings, increased PPT, and no effect in VDT and MDT. These results may suggest that the NSP and the various cupping therapies act in a similar way. 

The often reported deep relaxation induced by many of the manipulative and body-based therapies contributes to well-being and thus works through the affective-motivational component of pain [62] and probably adds to the overall treatment effect. 

There are several limitations in these studies. While there were no baseline differences and no drop outs in the BP study, a critical factor for the interpretation of the results of the NP study is the apparent baseline difference for NRS ratings, NPQ scores, QST-scores, and gender in the NP study. However, the analysis of covariance with the respective baseline value as a covariate controls in part for this difference. Moreover, the high percentage of female participants in both studies exceeds the gender-specific back or neck pain prevalences [9] likely suggesting that more female than male patients are interested in CAM treatments [63], therefore, the generalisability of the results to a male patient population is limited. 

The waiting list control strategy may be considered a potential weakness of the study. However, there is no valid inactive sham control for the needle stimulation pad available to date. Even the blunting of the sharp needle tips would still induce a rather strong mechanical stimulation. Therefore, a waiting list control seemed to be the best way to control for the effect of anticipation or for being included into a study. Furthermore, the waiting list control groups in these two studies are rather reflecting “standard treatment” since all patients were allowed to stay on their current treatment strategies, be it medication or physiotherapy, except for those treatments, which were among the exclusion criteria. Considering the fact that in clinical trials of conservative treatments for chronic nonspecific NP, changes in pain scores are generally similar between waiting-list control and placebo control groups [64], and the fact, that the sensory threshold measurements are not easy to see through for the study participants, we feel confident that the described treatment effects are valid. However, there are no good sham protocols for most these procedures available, although attempts to construct such controls are very innovative [65]. Blinding remains an issue to be debated on and furthermore, it was recently argued that, for example, sham acupuncture is associated with large unspecific effects [66]. This probably also accounts for any sham procedure for most of these interventions. Future clinical trials with a definite character should therefore compare different therapies with regard to their comparative effectiveness. 

In the two pilot studies presented here, a rather short-treatment protocol with defined treatment units was applied. However, in clinical practice, patients apply the pad typically whenever they feel intense pain and that may be several times a day. Until now no data are available, which treatment duration and frequency reveal the best therapeutic results. Because these studies were pilot studies, we used a pragmatic approach and did not change a protocol that derived from clinical experience, which means that participants pressed their hands (NP study) or their feet (BP study) in advance of the painful area. It is not clear if this protocol influences the outcome, but this question could be included in further trials. Nonetheless, it is doubtful that there is an exact dose-effect relationship, but more likely that there is an individual “optimal dose”.

## 6. Conclusion

The needle stimulation pad revealed a substantial potential for the alleviation of chronic NP and BP. Furthermore, psychophysical data support the assumption that the pad reveals its effects at least partly on a subcortical level of the pain processing system. A further benefit of the device is the fact that it is easy to use, safe, and does not require a therapist.

## Figures and Tables

**Table 1:** Baseline characteristics.

	NP study		BP study	
	Treatment group	Control group	Treatment group	Control group
*N*	17	18	21	21
Percentage female (%)	82.4	100	71.4	66.7
Mean (SD) age (years)	47.0 (10.9)	48.0 (12.1)	63.1 (11.9)	64.5 (10.6)

**Table 2:** Pre- to posttreatment changes (mean and standard deviation) and estimated group differences (from analysis of covariance) of outcome parameters. Bold values indicate significant *P* values.

	Control group		Needle stimulus pad		Estimated difference (95% CL)	*P*
	Pre	Post				
Pain (Numeric Rating Scale)						
Neck pain	4.4 (1.8)	4.5 (2.2)	4.9 (2.0)	3.4 (2.7)	−1.6 (−2.8 to − 0.3)	**0.021**
Lower back pain	4.9 (1.9)	5.4 (1.9)	5.0 (2.3)	3.2 (2.2)	−2.3 (−3.2 to − 1.3)	**<0.001**
Function (Neck Pain Questionnaire NPQ or Oswestry Disease Index ODI)						
Neck pain	33.2 (11.1)	32.5 (9.2)	36.3 (13.5)	26.5 (15.7)	−7.4 (−13.7 to − 1.1)	**0.028**
Lower back pain	25.0 (13.9)	19.9 (10.9)	22.8 (14.5)	18.8 (14.6)	0.4 (−4.8 to 5.6)	0.878
Log pressure pain threshold (PPT) area of maximum pain						
Neck pain	2.303 (0.168)	2.311 (0.182)	2.193 (0.221)	2.314 (0.264)	0.106 (0.013 to 0.198)	**0.032**
Lower back pain	2.548 (0.186)	2.534 (0.170)	2.552 (0.150)	2.619 (0.110)	0.082 (0.021 to 0.144)	**0.013**
Log pressure pain threshold (PPT) 10 cm close to maximum pain						
Neck pain	2.358 (0.173)	2.319 (0.142)	2.331 (0.202)	2.380 (0.264)	0.085 (−0.002 to 0.171)	0.064
Lower back pain	2.588 (0.178)	2.534 (0.170)	2.570 (0.138)	2.596 (0.109)	0.073 (0.006 to 0.139)	**0.038**
Log mechanical detection threshold (MDT) area of maximum pain						
Neck pain	0.118 (0.316)	0.174 (0.369)	0.129 (0.480)	0.132 (0.347)	−0.043 (−0.282 to 0.196)	0.726
Lower back pain	0.477 (0.598)	0.342 (0.526)	0.423 (0.375)	0.486 (0.413)	0.174 (−0.062 to 0.409)	0.156
Log mechanical detection threshold (MDT) 10 cm close to maximum pain						
Neck pain	−0.067 (0.410)	−0.080 (0.427)	0.128 (0.488)	−0.040 (0.422)	−0.049 (−0.306 to 0.208)	0.712
Lower back pain	0.563 (0.491)	0.376 (0.499)	0.317 (0.435)	0.359 (0.449)	0.145 (−0.085 to 0.376)	0.224
Vibration detection threshold (VDT) area of maximum pain						
Neck pain	6.7 (1.4)	6.7 (0.9)	6.3 (1.3)	6.1 (1.5)	−0.4 (−1.1 to 0.3)	0.247
Lower back pain	3.8 (2.0)	4.1 (1.7)	4.2 (1.7)	4.2 (1.9)	−0.2 (−1.0 to 0.5)	0.514
Vibration detection threshold (VDT) 10 cm close to maximum pain						
Neck pain	6.5 (1.0)	6.5 (1.1)	6.0 (1.4)	5.7 (1.5)	−0.5 (−1.1 to 0.2)	0.165
Lower back pain	5.1 (1.1)	5.0 (1.3)	5.0 (1.6)	5.0 (1.2)	0.1 (−0.5 to 0.7)	0.774

**Table 3:** Spearman correlation coefficients for detections thresholds in control areas.

	Neck pain		Lower back pain	
	Hand	Foot	Hand	Foot
PPT	0.833 (*P* < 0.001)	0.690 (*P* < .001)	0.601 (*P* < .001)	0.590 (*P* < 0.001)
MDT	0.752 (*P* < 0.001)	0.568 (*P* < .001)	0.686 (*P* < .001)	0.632 (*P* < 0.001)
VDT	0.541 (*P* = 0.001)	0.645 (*P* < .001)	0.420 (*P* = .006)	0.740 (*P* < 0.001)
